# Integrated xTB and simplified Tamm–Dancoff analysis of composition-dependent electronic structure in GaInZnP/ZnSe_y_S_1-y_ core/shell quantum dots with DFT and TDDFPT benchmarking

**DOI:** 10.1007/s00894-026-06728-1

**Published:** 2026-05-08

**Authors:** Oluwasesan Adegoke, Ojodomo J. Achadu

**Affiliations:** 1 Leverhulme Research Centre for Forensic Science, Faculty of Science, Engineering and Business, University of Dundee, Dundee, DD1 4HN UK; 2 School of Health and Life Sciences, National Horizon Centre, Teesside University, Middlesbrough, TS1 3BA UK

**Keywords:** Quantum dots, DFT, Electronic structure, xTD, sTDA, TDDFPT

## Abstract

**Context:**

Alloyed core/shell quantum dots (QDs) provide a platform for composition-controlled modulation of electronic structure and optical response. Using an integrated extended tight binding (xTB) and simplified Tamm Dancoff approximation (sTDA) framework, a composition-defined GaInZnP/ZnSe_y_S_1-y_ core/shell QD series (*y* = 0.00, 0.25, 0.50, 0.75, 1.00) revealed a distinctly nonlinear dependence of electronic and excited-state behaviour on shell composition. Relative stability favoured S-rich shells, while mixed-shell compositions showed non-ideal energetic behaviour with a pronounced deviation at *y* = 0.75. The frontier electronic structure did not vary monotonically with Se content, while the optical response showed composition-dependent redistribution of low-energy transitions. Benchmark comparison with density functional theory (DFT) and time-dependent density functional perturbation theory (TDDFPT) confirmed that the nonlinear evolution of frontier-level separation and absorption behaviour was an intrinsic feature of the alloy series, although the exact magnitude and position of the extrema remain method dependent. A distinct anomaly at *y* = 0.75 indicated enhanced frontier state reorganisation within the mixed-shell environment.

**Method:**

Atomistic GaInZnP/ZnSe_y_S_1-y_ QDs spanning *y* = 0.00 to 1.00 were analysed under identical structural constraints to isolate shell anion substitution effects. Ground-state electronic structure and excited-state properties were evaluated using xTB and sTDA, with DFT and TDDFPT used as independent benchmarks for frontier electronic structure and optical absorption trends. Composition-dependent changes in energetic stability, frontier-orbital separation, density-of-states like distributions, absorption onset, and frontier-orbital localisation were then compared across the full alloy series using a consistent analysis framework.

**Supplementary Information:**

The online version contains supplementary material available at 10.1007/s00894-026-06728-1.

## Introduction

Semiconductor quantum dots (QDs) are nanoscale materials whose electronic and optical properties are governed by quantum confinement, atomistic composition, and interfacial structure [1, 2]. These characteristics have established QDs as key materials for applications in light emission, photodetection, bioimaging, and quantum technologies [3]. In such systems, small variations in composition or structure can produce pronounced changes in electronic structure and excited-state behaviour, making composition-controlled design a central challenge in QDs research [1, 2]. Core/shell architecture provides a robust strategy for tuning QD properties by combining materials with distinct electronic characteristics within a single nanostructure [4–6]. By surrounding a semiconductor core with a compositionally engineered shell, carrier confinement, interfacial electronic structure, and optical transition probabilities can be modulated in a controlled manner [6]. Alloyed shells further expand the design space by enabling continuous adjustment of electronic properties without altering particle size [4]. Among these systems, GaInZnP-based cores are of particular interest due to their cadmium-free composition and tunable electronic structure [7, 8], while ZnSe-based and ZnS-based shells offer favourable band alignment and chemical stability [9–11]. In ZnSe_y_S_1-y_ alloy shells, systematic variation of the Se to S ratio can modify band offsets, dielectric screening, and confinement potentials experienced by charge carriers [10]. These changes influence both the energetic position of excited states and their optical activity, as reflected in transition wavelengths and oscillator strengths. Although experimental studies have reported composition-dependent optical trends in such systems, direct interpretation at the electronic structure level is complicated by the presence of size dispersion, surface effects, and structural heterogeneity inherent to colloidal synthesis [12].

Computational modelling provides a direct route to disentangling composition-driven effects by enforcing strict control over structure, stoichiometry, and atom count [13]. However, a comprehensive theoretical description of excited states in QDs remains challenging due to the large system sizes required to capture realistic core/shell architectures [13]. High-level first-principles methods, while accurate, are often prohibitively expensive for systematic exploration across multiple shell compositions, which limits their use in composition-resolved studies [13]. In this context, the extended tight-binding (xTB) electronic structure method and the simplified Tamm–Dancoff approximation (sTDA) provide an efficient and internally consistent computational workflow for investigating electronic-structure and excited-state properties in nanoscale systems [14–18]. In the present work, xTB was used directly as the semi-empirical ground-state electronic-structure method, rather than as the basis for a separately derived tight-binding model developed within this study. The xTB calculations provided the optimised geometries, orbital energies, and molecular-orbital reference used throughout the analysis [14–16]. This reference electronic structure was then used as the input for the subsequent sTDA excited-state calculations [17, 18]. The combined workflow enabled systematic comparison of composition-dependent trends while maintaining identical structural constraints and numerical treatment across the full series [19].

To ensure that the observed electronic structure trends are physically meaningful and not artefacts of the semi-empirical framework, independent first-principles validation was incorporated through density functional theory (DFT) and time-dependent density functional perturbation theory (TDDFPT). DFT was used to evaluate ground-state electronic structure and frontier-orbital separation within a plane-wave pseudopotential framework, while TDDFPT was employed to compute frequency-dependent optical response and absorption spectra based on the converged ground-state charge density [20]. This combined approach can enable direct comparison between the xTB sTDA-derived electronic and optical descriptors and those obtained from first-principles methods, thereby establishing the reliability of the observed composition-dependent trends across multiple theoretical levels [21]. Importantly, this validation strategy focuses on consistency of trends rather than absolute agreement, given the known differences in energy scale and excitation description between semi-empirical and first-principles methods. Within alloyed QD core/shell systems, the electronic structure response to compositional variation is often assumed to follow a monotonic interpolation between end-member materials. However, deviations from this behaviour can arise from local chemical environment effects, orbital mixing, and changes in interfacial electronic coupling [22]. Resolving such behaviour requires a computational framework in which structure, atom count, and numerical treatment are strictly controlled, allowing composition to be isolated as the sole variable. In this context, the present study establishes a composition-resolved analysis of GaInZnP/ZnSe_y_S_1-y_ core/shell QDs in which all structural degrees of freedom are fixed across the full alloy range, enabling direct attribution of electronic and excited-state variations to shell anion substitution.

In this work, a controlled series of GaInZnP/ZnSe_y_S_1-y_ core/shell QDs is investigated using an integrated xTB and sTDA computational approach. This approach represents a consistent computational framework in which both methods are applied sequentially to the same electronic structure reference, rather than a new parameterised or unified theoretical model. All models share identical geometry, atom count, and core composition, ensuring that observed variations in electronic structure and excited-state properties arise solely from changes in shell composition. Excitation energies, transition wavelengths, and oscillator strengths are analysed for optically active excited states across the full alloy range, providing a coherent and internally consistent picture of how shell composition governs excited-state behaviour in alloyed core/shell QDs. The integration of xTB sTDA with DFT and TDDFPT benchmarking provides a unified interpretation of composition-dependent electronic structure and optical response. This framework enables identification of both monotonic and non-monotonic trends in frontier orbital separation, density of states (DOS) distributions, and absorption behaviour, while maintaining strict consistency in structural representation. The results therefore provide a validated theoretical basis for understanding composition-driven electronic structure evolution in alloyed core/shell QDs and establish a robust platform for guiding composition-controlled design of nanoscale semiconductor systems.

## Computational methods

### Construction of GaInZnP/ZnSe_y_S_1-y_ core/shell QDs models

All calculations were performed on atomistic models of GaInZnP/ZnSe_y_S_1-y_ core/shell QDs, constructed under strictly controlled geometric and compositional constraints, to enable systematic comparison across shell compositions. The shell composition was varied as ZnSe_y_S_1-y_, with *y* values of 0.00, 0.25, 0.50, 0.75, and 1.00. For all models, the core radius was fixed at 6.0 Å, and the shell thickness was fixed at 1.5 Å. No surface ligands, passivating species, or solvent effects were included. The QDs were therefore modelled with unpassivated surfaces to isolate the intrinsic influence of shell anion substitution under identical structural constraints across the series. The absence of surface passivation introduces dangling bond states and surface-localised electronic states that can influence the absolute magnitude of the HOMO–LUMO gap and low-energy optical transitions; therefore all electronic and optical quantities are interpreted as internal composition-dependent trends within a fixed structural model rather than as direct experimental values. All atomic configurations were generated and managed using the Atomic Simulation Environment (ASE), with a fixed random seed, to ensure reproducibility, and to eliminate stochastic structural variability between compositions.

#### Ground state electronic structure calculations

Ground-state electronic-structure calculations were performed using xTB as the direct semi-empirical electronic-structure method employed in this work. No separate tight-binding Hamiltonian or reduced model was derived from the xTB output. Instead, xTB was used directly to obtain the optimised geometries, orbital energies, and molecular orbital information for each QD composition. All electronic-structure calculations were executed through the ASE interface to ensure consistent structure handling and workflow control. For each QD composition, identical numerical settings and convergence criteria were applied. Geometry optimisation of all QD models was performed using GFN2 xTB, and the resulting optimised geometries, xtbopt.xyz, were used as the reference structures for all subsequent electronic-structure and excited-state calculations. To provide an independent validation of electronic-structure trends, DFT calculations were performed using Quantum ESPRESSO within a plane-wave pseudopotential framework. The Perdew–Burke–Ernzerhof (PBE) exchange-correlation functional was used. A kinetic energy cutoff of 20 Ry and a charge-density cutoff of 160 Ry were applied. Electronic occupations were treated using Gaussian smearing with a degauss value of 0.01 Ry. All calculations were performed at the Gamma point, consistent with finite-size QD models. Self-consistent field (SCF) calculations were carried out for each composition to obtain converged ground-state charge densities and total energies. Non-SCF (NSCF) calculations were subsequently performed using the converged SCF charge density to obtain an accurate description of the unoccupied electronic states required for DOS analysis.

#### Excited-state calculations using simplified Tamm–Dancoff approximation

Electronic excited states were computed using the sTDA, applied to the xTB ground-state electronic-structure reference. In this workflow, the molecular orbitals and orbital energies obtained from xTB defined the electronic structure basis used for all excited-state calculations. The sTDA was implemented within the sTDA framework by restricting the excitation space to single-electron transitions from occupied to unoccupied orbitals while neglecting the corresponding de-excitation coupling terms. This treatment reduces computational cost and improves numerical stability for systems with large atom counts, while retaining the dominant contributions to low-energy optical transitions. The role of xTB was therefore to provide a consistent ground-state electronic-structure reference, while sTDA evaluates excitation energies, transition wavelengths, and oscillator strengths within this restricted excitation space. The connection between the two methods is thus a sequential and internally consistent computational procedure rather than a derived tight-binding formalism. Orbital onset energy was defined as the energy of the lowest unoccupied molecular orbital (LUMO) following alignment of all orbital energies to the highest occupied molecular orbital (HOMO) reference (*E* = 0 eV). Orbital energies were taken directly from xTB single-point calculations on the optimised geometries. The onset therefore represented the first discrete unoccupied frontier level within the model rather than an optically allowed excitation energy. Core and shell orbital-localisation fractions were obtained by integrating |ψ|^2^ contributions over atoms belonging to the predefined core and shell regions. The core region was defined as the GaInZnP framework, and the shell region as the ZnSe_y_S_1-y_ outer layer, consistent with the structural model described in the previous section. Orbital coefficients were extracted from xTB single-point calculations, and atomic contributions were summed to obtain normalized core and shell fractions for each frontier orbital. To further resolve the electronic structure obtained from DFT, DOS calculations were performed using the dos.x module, and projected DOS (PDOS) calculations were performed using projwfc.x. These calculations enabled decomposition of the electronic states into element-resolved contributions, allowing direct comparison with HOMO–LUMO gap trends and E_HOMO_ aligned DOS distributions.

#### Identification of optically active excited states

To ensure physically meaningful analysis of optical trends, a uniform selection criterion was applied across all compositions. For each shell composition, the lowest-energy excited state with nonzero oscillator strength was identified and defined as the lowest bright excited state. This state was used to define the lowest bright transition energy for each composition, while the full set of optically allowed excited states was retained to analyse the distribution of excitation energies and oscillator strengths across the accessible energy range. All excited state data were consolidated into a single structured table, which served as the sole source of data for downstream quantitative analysis and figure generation. The dataset therefore supports both lowest-energy transition analysis and distribution-based evaluation of excited states as a function of excitation energy and oscillator strength. In addition to sTDA-based optical analysis, optical absorption spectra were calculated using TDDFPT as implemented in Quantum ESPRESSO. These calculations were performed using the turbo TDDFPT module based on the converged SCF ground state [23–25]. A Lanczos recursion scheme was employed with a fixed number of iterations to ensure consistent spectral resolution across all compositions. The frequency-dependent polarizability was computed over an energy range of 0 to 8 eV, and absorption spectra were obtained directly from the imaginary component of the polarizability. Absorption onset energies were determined from the TDDFPT spectra using a fixed intensity threshold criterion, defined as the lowest energy at which the absorption intensity exceeds a specified fraction of the maximum intensity. This procedure was applied consistently across all compositions to enable direct comparison of absorption onset trends.

#### Data processing, visualisation, and software implementation

Post-processing, analysis, and visualisation of electronic and excited-state data were performed using custom Python scripts executed within the ASE workflow environment. Numerical analysis and plotting were carried out using standard scientific Python libraries, including NumPy and Matplotlib. All figures were generated directly from tabulated excited-state data, without manual adjustment. All calculations were executed within a Linux environment, using Windows Subsystem for Linux. All software packages used in this work, including ASE, xTB, sTDA, Python, NumPy, and Matplotlib, are open access, and freely available to the scientific community.

## Results and discussion

### Atomistic structure of GaInZnP/ZnSe_y_S_1-y_ QDs

**Fig. 1:**
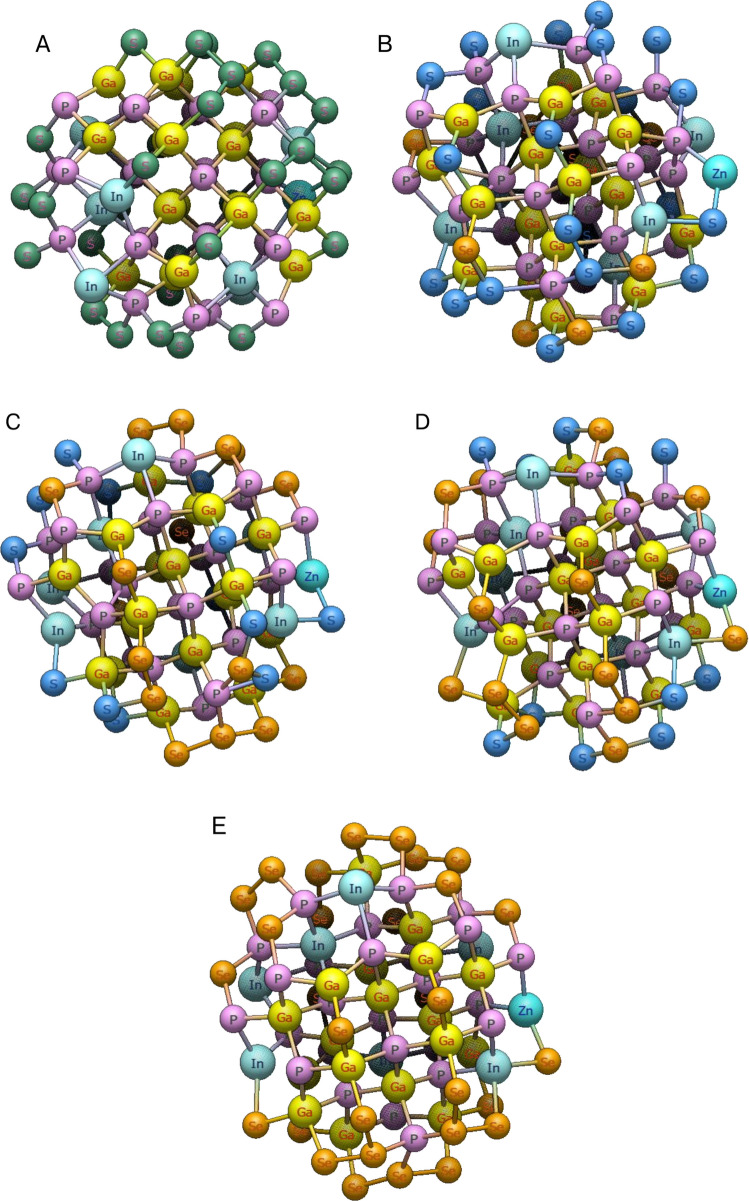
Atomistic structures of the GaInZnP/ZnSe_y_S_1-y_ core/shell QDs for **A** *y* = 0.00, GaInZnP/ZnS; **B** *y* = 0.25, GaInZnP/ZnSe_0.25_S_0.75_; **C** *y* = 0.50, GaInZnP/ZnSe_0.50_S_0.50_; **D** *y* = 0.75, GaInZnP/ZnSe_0.75_S_0.25_; **E** *y* = 1.00, GaInZnP/ZnSe

The GaInZnP/ZnSe_y_S_1-y_ core/shell QDs were constructed using a fixed atomistic framework in which the core composition, size, and connectivity were preserved while the shell anion composition was systematically varied. This approach enabled direct isolation of compositional effects arising from S to Se substitution in the shell, while eliminating geometric or configurational variability across the series. Figure 1 shows the relaxed structures of the QDs spanning the full composition range defined by the general formula GaInZnP/ZnSe_y_S_1-y_, with *y* = 0.00, 0.25, 0.50, 0.75, and 1.00. All QDs share the same total atom count, radial dimensions, and bonding topology, ensuring that each structure differs only in the chemical identity of the shell anions. Figure 1A corresponds to *y* = 0.00 (GaInZnP/ZnS), where S fully occupies the shell region surrounding the GaInZnP core. The structure exhibits a compact and radially symmetric core/shell arrangement, with the cation sublattice remaining unchanged throughout the particle. This configuration serves as the S-rich end member of the compositional series. Figure 1B, on the other hand, shows the *y* = 0.25 composition (GaInZnP/ZnSe_0.25_S_0.75_), in which Se is introduced into the shell while preserving the same spatial framework. Se substitution occurs uniformly across shell sites, producing a chemically mixed shell without disrupting the underlying atomic connectivity. Figure 1C corresponds to *y* = 0.50, (GaInZnP/ZnSe_0.50_S_0.50_), representing an equal S and Se shell composition. The QD retained its original size and morphology, providing a chemically balanced reference structure for evaluating composition-driven trends in subsequent electronic and optical analysis. Figure 1D illustrates the *y* = 0.75 system, (GaInZnP/ZnSe_0.75_S_0.25_), where Se becomes the dominant shell anion. The progressive substitution proceeds without inducing structural distortion, indicating that the chosen model accommodates continuous compositional variation without strain driven reconstruction. Figure 1E shows the *y* = 1.00 composition, (GaInZnP/ZnSe), in which the shell consists entirely of Se. Despite the complete anion substitution, the core/shell geometry and atomic topology remain consistent with the rest of the series, confirming the robustness of the structural framework. Across all five QDs, the structural invariance ensured that differences observed in electronic structure and excited-state behaviour can be attributed exclusively to shell composition. The computational procedure used for extraction, validation, and standardisation of the optimised atomic structures is provided in Fig. S1, which outlines the structure processing workflow and corresponding minimal implementation.

### Relative stability and compositional energetics of GaInZnP/ZnSe_y_S_1-y_ core/shell QDs

**Fig. 2:**
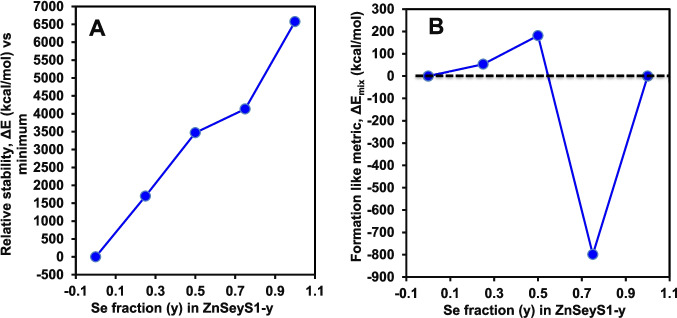
Relative stability and compositional energetics of GaInZnP/ZnSe_y_S_1-y_ core/shell QDs showing the relative stability Δ*E* as a function of Se fraction *y* in the ZnSe_y_S_1-y_ shell, referenced to the lowest energy composition in the series. **B** Mixing deviation Δ*E*_mix_ is defined as the deviation of the total energy from linear interpolation between *y* = 0.00 and 1.00 end-member compositions within the fixed-atom-count model

Figure 2 examines the energetic response of the GaInZnP/ZnSe_y_S_1-y_ core/shell QDs to systematic variation in shell composition, providing insight into relative stability and compositional energetics within a strictly controlled structural framework. Figure 2A shows the relative stability, expressed as the Δ*E* with respect to the most stable composition in the series from xTB calculations. A clear monotonic increase in relative energy is observed as the Se fraction *y* increased from 0.00 to 1.00. The ZnS-shelled QDs at *y* = 0 define the energetic minimum, while progressive substitution of S by Se leads to increasingly higher total energies. This trend indicates that, within the chosen atomistic model and electronic structure reference, S-rich shells are energetically favoured over Se-rich shells. Importantly, the smooth nature of the curve reflects the absence of structural discontinuities or numerical artefacts, confirming that the observed behaviour arises from intrinsic chemical substitution effects rather than changes in geometry or atomic arrangement [26].

Figure 2B presents the mixing deviation Δ*E*_mix_ from xTB calculations, defined as the difference between the total energy of each composition and the linearly interpolated energy between *y* = 0.00 and *y* = 1.00 end member compositions within the fixed atom count model. This quantity represents the degree of nonideal energetic behaviour associated with shell alloying within the model rather than a thermodynamic formation energy referenced to external reservoirs. The *y* = 0.25 and *y* = 0.50 compositions exhibit positive deviations, showing positive deviation from the linear end member interpolation within this fixed size model. In contrast, the *y* = 0.75 composition shows a pronounced negative deviation, corresponding to a negative deviation from the linear end member interpolation within this fixed size model. This behaviour suggests a nonlinear energetic response to shell alloying, with specific intermediate compositions benefiting from favourable local chemical environments within the shell [27]. Notably, the *y* = 1.00 composition returned to a value close to the linear reference, indicating that the energetic anomaly is confined to intermediate Se rich mixed shells rather than being a general feature of Se substitution. The presence of a distinct minimum in the mixing metric at *y* = 0.75 highlights that compositional effects in alloyed shells cannot be inferred solely from end member energetics. Together, Fig. 2A and B demonstrate that while the overall energetic preference favours S rich shells, the internal energetics of mixed S-Se shells are non-trivial and composition dependent. Because energies are referenced only within the fixed atom count series and not to external chemical reservoirs, Δ*E* and Δ*E*_mix_ are used here as comparative model internal metrics rather than formation energies. The workflow used for evaluation of relative stability and mixing behaviour, including calculation of Δ*E* and Δ*E*_mix_ from total energies under consistent numerical treatment, is provided in Fig. S2.

### Shell-composition-dependent evolution of the frontier electronic structure

**Fig. 3:**
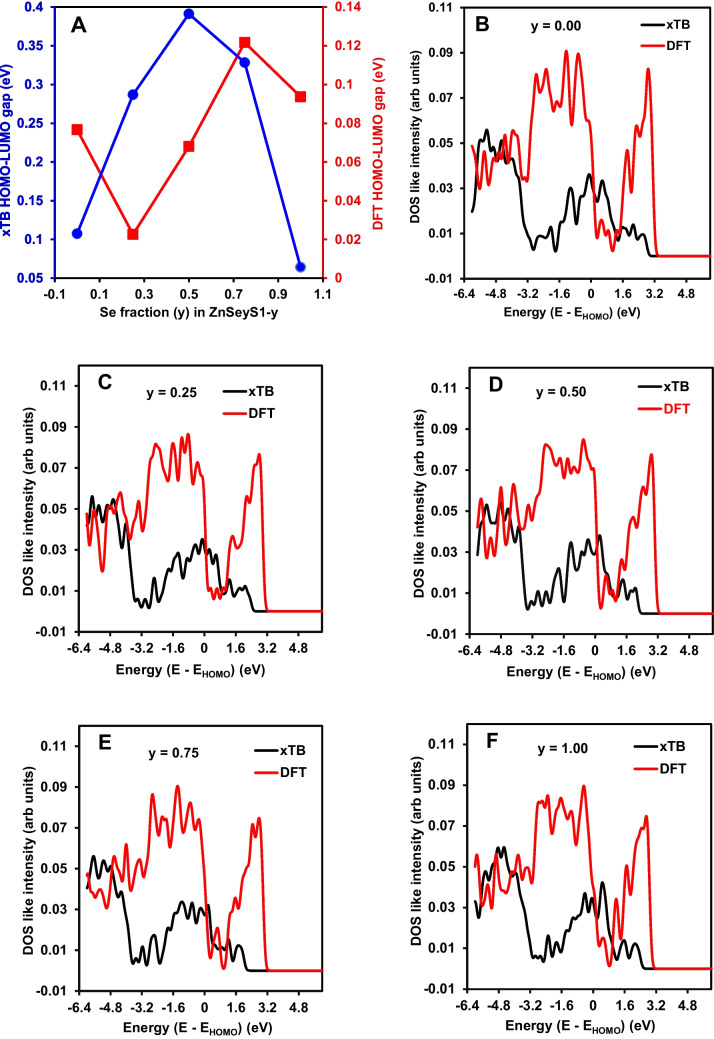
Composition-dependent electronic structure of GaInZnP/ZnSe_y_S_1-y_ core/shell QDs showing **A** xTB and DFT HOMO–LUMO gap as a function of the Se fraction *y* in the ZnSe_y_S_1-y_ shell and **B**–**F**) E_HOMO_ aligned DOS like distributions showing xTB molecular-orbital-derived spectra with DFT benchmark overlay for **B** *y* = 0.00, **C** 0.25, **D** 0.50, **E** 0.75, and **F** 1.00, respectively

Figure 3A shows the variation of the xTB HOMO–LUMO gap as a function of the Se fraction *y* in the ZnSe_y_S_1-y_ shell. The dependence is distinctly nonlinear, in which the gap increases from the S-rich composition *y* = 0.00 to a maximum at the intermediate composition *y* = 0.50, followed by a marked decrease toward the Se-rich limit *y* = 1.00. This behaviour demonstrates that anion alloying in the shell does not lead to a monotonic interpolation between ZnS and ZnSe endpoints but instead gives rise to a pronounced extremum at intermediate composition [28, 29]. Figure 3B–F presents the DOS-like distributions for each composition, aligned with respect to the corresponding E_HOMO_. Across the full compositional range, the spectra retain a comparable overall structure, characterised by a dominant occupied manifold at negative energies and structured intensity extending into the near-frontier region. This similarity indicates that the fundamental electronic architecture of the GaInZnP-based core/shell system is preserved as *y* varies, while the shell composition modulates the detailed arrangement of molecular orbital levels around the frontier. The DOS-like spectra further revealed a composition-dependent redistribution of spectral weight in the near-frontier region, where increasing Se fraction leads to a broader distribution of states and enhanced fine structure. These changes reflect variations in orbital mixing and level spacing induced by anion substitution within the shell, rather than the emergence of new electronic manifolds [28]. At the intermediate composition *y* = 0.50, as shown in Fig. 3D, the DOS-like spectrum remains qualitatively consistent with those at other compositions, yet the corresponding HOMO–LUMO gap in Fig. 3A reaches its maximum, indicating that the enhanced gap originates from the relative positioning of discrete frontier orbitals rather than a reorganisation of the overall electronic structure. Similarly, the reduced gap at *y* = 1.00 is accompanied by a DOS-like spectrum in Fig. 3F, which preserves the same general features but reflects a different frontier level spacing. The HOMO–LUMO gap is therefore interpreted as a descriptor of frontier orbital spacing within the model rather than a direct measure of optical absorption onset. In addition, the absence of surface passivation means that the frontier orbital energies include contributions from surface-localised states, so the absolute gap values are not representative of fully passivated or experimentally observed quasiparticle gaps.

Figure 3A further includes DFT HOMO–LUMO gaps extracted from the NSCF calculations, which are consistently smaller in magnitude across all compositions and exhibit a nonlinear dependence on shell composition. In contrast to the xTB result, the DFT gap reaches its maximum at *y* = 0.75 before decreasing at *y* = 1.00. This shift in the position of the extremum indicates sensitivity of the quantitative gap description to the electronic structure formalism, while the preservation of non-monotonic behaviour across both methods confirms that the composition-dependent evolution of frontier level separation is intrinsic to the system. The reduced DFT gap values are consistent with the combined influence of PBE gap underestimation and the presence of surface-related states in the unpassivated QD models, both of which act to compress the frontier level separation. The DFT benchmark DOS-like distributions shown in Fig. 3B–F exhibit the same overall spectral organisation observed in the xTB results, with a dominant occupied manifold at negative energies and structured intensity approaching the frontier region. Although the DFT spectra displayed broader features and reduced apparent gaps, the relative arrangement of states and the progressive redistribution of near-frontier intensity with increasing Se content are preserved. Alignment with respect to E_HOMO_ enables direct comparison between the two methods and demonstrates that the composition-dependent modification of frontier electronic structure is consistently captured. The combined analysis shows that variations in the HOMO–LUMO gap arise primarily from shifts in the energies of frontier orbitals, while the broader electronic manifold remains largely unchanged across the composition series.

In general, Fig. 3A–F shows that ZnSe_y_S_1-y_ shell alloying primarily governs the separation of frontier electronic levels while maintaining the overall electronic framework of the system. DOS-like spectra capture composition-dependent redistribution of molecular orbital density relative to E_HOMO_, whereas the HOMO–LUMO gap provides a quantitative measure of the evolution of frontier level separation. The combined xTB and DFT analysis established that the nonlinear dependence of the frontier gap on shell composition is a robust feature of the system, with differences between methods confined to quantitative values and the exact composition of the extremum rather than the underlying physical trend. The appearance of a maximum gap at *y* = 0.50 highlights the nontrivial role of mixed anion shells in tuning the electronic structure of GaInZnP/ZnSe_y_S_1-y_ core/shell QDs [30], while the DFT benchmark confirms that the tuning remains nonlinear when evaluated with an independent electronic structure method. The procedure used for determination of HOMO–LUMO gaps and construction of DOS-like distributions from molecular orbital energies is provided in Fig. S3. It is important to note that the HOMO–LUMO gap represents a discrete orbital energy difference and should be distinguished from orbital onset energy, which reflects the effective onset of the unoccupied manifold after alignment.

### Composition-dependent optical response of GaInZnP/ZnSe_y_S_1-y_ core/shell QDs

**Fig. 4:**
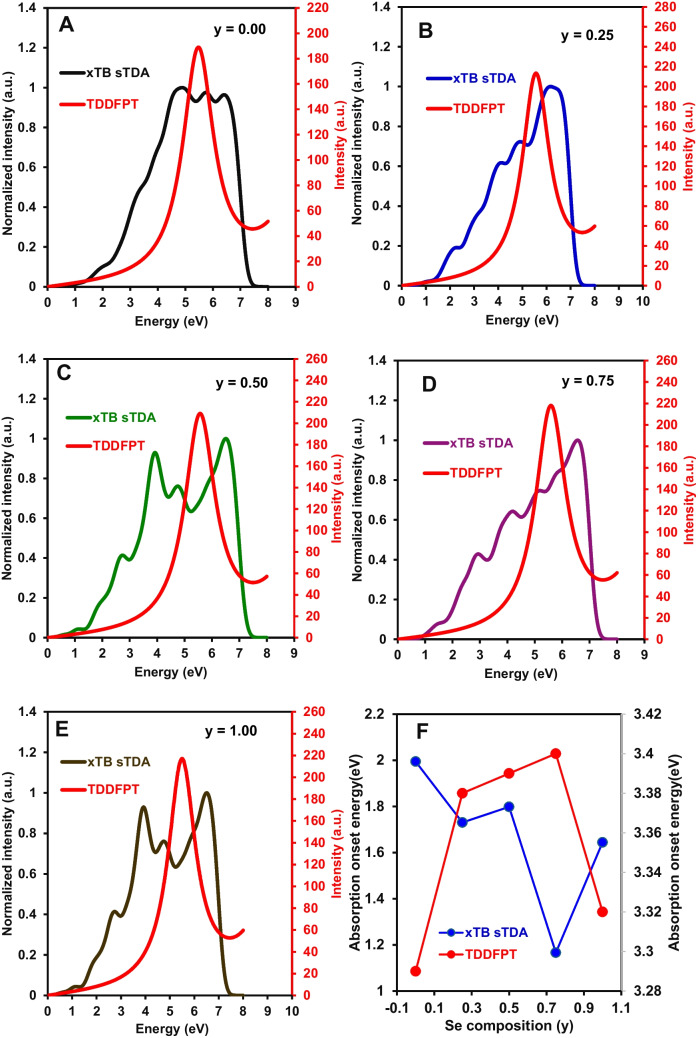
Composition-dependent optical absorption of GaInZnP/ZnSe_y_S_1-y_ core/shell QDs showing absorption spectra for shell compositions **A** *y* = 0.00, **B** 0.25, **C** 0.50, **D** 0.75, and **E** 1.00, plotted as a function of photon energy using xTB sTDA and TDDFPT. **F** Absorption onset energy as a function of shell composition *y* obtained from xTB sTDA and TDDFPT

The composition-dependent optical response of GaInZnP/ZnSe_y_S_1-y_ core/shell QDs was analysed using xTB sTDA absorption-like spectra, followed by TDDFPT calculations to provide a higher-level description of the dominant excited-state transitions. The combined analysis enabled a consistent evaluation of both spectral shape and transition energetics across the full compositional range. Figure 4A–E shows the absorption spectra obtained using xTB sTDA for increasing Se fraction *y*. All compositions exhibit broad absorption bands, with systematic variation in spectral structure. For the S-rich composition *y* = 0.00 in Fig. 4A, the absorption profile is broad with limited internal structure and a gradual increase in intensity toward higher energy, indicating a relatively narrow distribution of optically allowed transitions. At *y* = 0.25 in Fig. 4B, additional structure emerges, with discernible shoulders at intermediate energies and a slight extension of spectral weight toward lower energy. For *y* = 0.50 in Fig. 4C, multiple local features are resolved across the absorption band, indicating a broader distribution of transition energies without a dominant redistribution of total intensity. At *y* = 0.75 in Fig. 4D, the spectral weight shifts further toward lower energy, with increased prominence of the low-energy region and reduced relative contribution at higher energy. For the Se-rich composition *y* = 1.00 in Fig. 4E, the spectrum remains broad and structured, with partial redistribution of intensity toward higher energy relative to *y* = 0.75. These results show that shell composition modified both the distribution and relative contribution of optically allowed transitions across the spectrum. The absorption onset extracted from the xTB sTDA spectra is shown in Fig. 4F. The onset energy decreased from *y* = 0.00 to *y* = 0.75 (an increase was observed at *y* = 0.50) and increases at *y* = 1.00, demonstrating a nonlinear dependence on shell composition. This behaviour reflects modification of the low-energy transition manifold rather than a uniform shift of the entire absorption band. The unpassivated surface also affects low-energy optical transitions through surface-related states; therefore, the absorption onset energies reported here reflect the internal behaviour of the model system under fixed structural conditions rather than absolute experimental onset values.

The TDDFPT results provide a benchmark description of the dominant absorption feature and are first evaluated using the compositional overlay in Fig. S4. The overlay shows a single intense absorption band for all compositions within the 5–6 eV region, while the inset resolves the relative peak positions and intensities. The *y* = 0.00 spectrum shows the lowest peak energy and lowest intensity within the series. With Se incorporation, the peak shifts slightly to higher energy for *y* = 0.25 and *y* = 0.50 and reaches the highest energy and highest intensity at *y* = 0.75. At *y* = 1.00, the peak shifts to lower energy relative to *y* = 0.75 and exhibits a reduction in intensity while remaining higher than the S-rich composition. In comparison with the xTB sTDA results, TDDFPT yields a more localised and well-defined principal absorption feature (Fig. 4A–E), whereas the xTB sTDA spectra showed broader distributions with multiple overlapping contributions. This difference indicates that xTB sTDA captures the overall redistribution of transition density across the energy range, while TDDFPT resolves the dominant excitation with greater clarity. Despite these differences in spectral representation, both approaches consistently show that the optical response does not vary monotonically with composition and that intermediate Se fractions play a key role in determining transition energy and intensity. The TDDFPT absorption onset values extracted in Fig. 4F also followed a consistent nonlinear trend. The TDDFPT onset increases from *y* = 0.00 to *y* = 0.75, where the highest onset energy is observed, and then decreases at *y* = 1.00. In contrast to the xTB sTDA onset, which shows a decrease to *y* = 0.75 followed by an increase, the TDDFPT onset shows the opposite trend in magnitude while retaining the same nonlinear dependence on composition. This difference reflects the distinct treatment of excited states in the two approaches, with TDDFPT providing a more direct description of the principal transition energy. It is important to emphasise the absence of direct correlation between the HOMO–LUMO gap in Fig. 3A and the absorption onset energy in Fig. 4F arises because the HOMO–LUMO gap reflects frontier orbital energy separation including surface-localised states, whereas the absorption onset corresponds to the lowest energy optically allowed transition determined by oscillator strength, and therefore probes a different physical quantity.

In general, the xTB sTDA and TDDFPT results consistently showed that ZnSe_y_S_1-y_ shell composition governs the optical response of GaInZnP core/shell QDs through nonlinear modulation of transition energy and spectral distribution. While xTB sTDA captures the broad spectral evolution and redistribution of absorption features, TDDFPT provides a more resolved description of the dominant transition and its compositional dependence. Both approaches show that the most pronounced deviation from monotonic behaviour occurs at the intermediate composition *y* = 0.75, although the direction of this deviation differs between the two methods, with xTB sTDA showing a minimum in absorption onset and TDDFPT showing a maximum at this composition. This behaviour indicates that mixed anion shells generate a distinct excited-state distribution that cannot be described as a linear interpolation between ZnS and ZnSe limits. The workflow used for the construction of the absorption spectra and extraction of absorption onset energy from excited-state data is provided in Fig. S5.

### Composition dependent evolution of optical descriptors and excited state distributions

Figure S6A summarises the evolution of the absorption onset energy and the dominant absorption peak energy as a function of the Se fraction *y* in the ZnSe_y_S_1-y_ shell from xTB calculations [31]. The absorption onset energy shows a non-monotonic dependence on y, decreasing from *y* = 0.00 to a minimum at *y* = 0.75 (a slight increase was observed at *y* = 0.50), followed by an increase at *y* = 1.00. In contrast, the peak energy increased from *y* = 0.00 to intermediate compositions and then varies more modestly toward higher y. These trends show that the onset energy and peak energy respond differently to changes in *y* across the series. Figure S6B presents the distribution of individual excited states plotted as oscillator strength (f) versus excitation energy for each composition also from xTB calculations [17]. For *y* = 0.00, the spectrum contains relatively few transitions with appreciable f in the lower excitation energy region, with most transitions remaining weak. At *y* = 0.25 and *y* = 0.50, additional transitions with non-negligible f appear across the low to intermediate excitation energy range, and the distribution of sticks becomes more populated relative to *y* = 0.00. For *y* = 0.75, Figure S6B contains the most prominent high f transition in the plotted range and multiple additional transitions with appreciable f at intermediate to higher excitation energies. At *y* = 1.00, transitions with appreciable f remain present across a similar excitation energy window, while the distribution of oscillator strengths differs from *y* = 0.75. Figure S6A and S6B shows that varying *y* modulates both the energetic descriptors of the absorption spectrum and the distribution of optically allowed excited states. The non-monotonic trend in absorption onset energy in Fig. S6A is accompanied by composition-dependent differences in the excitation energy and oscillator strength distributions in Fig. S6B, particularly at *y* = 0.75 where the most intense transition is observed. The algorithmic procedure used for the extraction of optical descriptors and construction of oscillator strength distributions across the compositions is provided in Fig. S7.

### Composition dependent frontier orbital spatial distributions

**Fig. 5:**
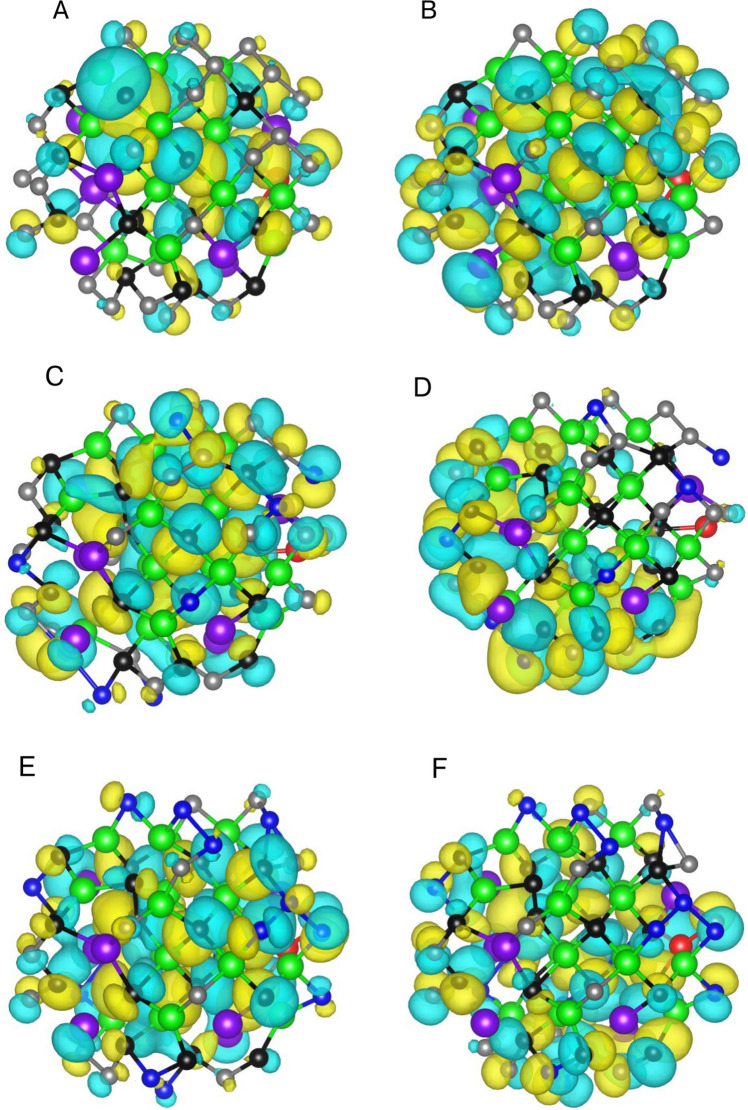

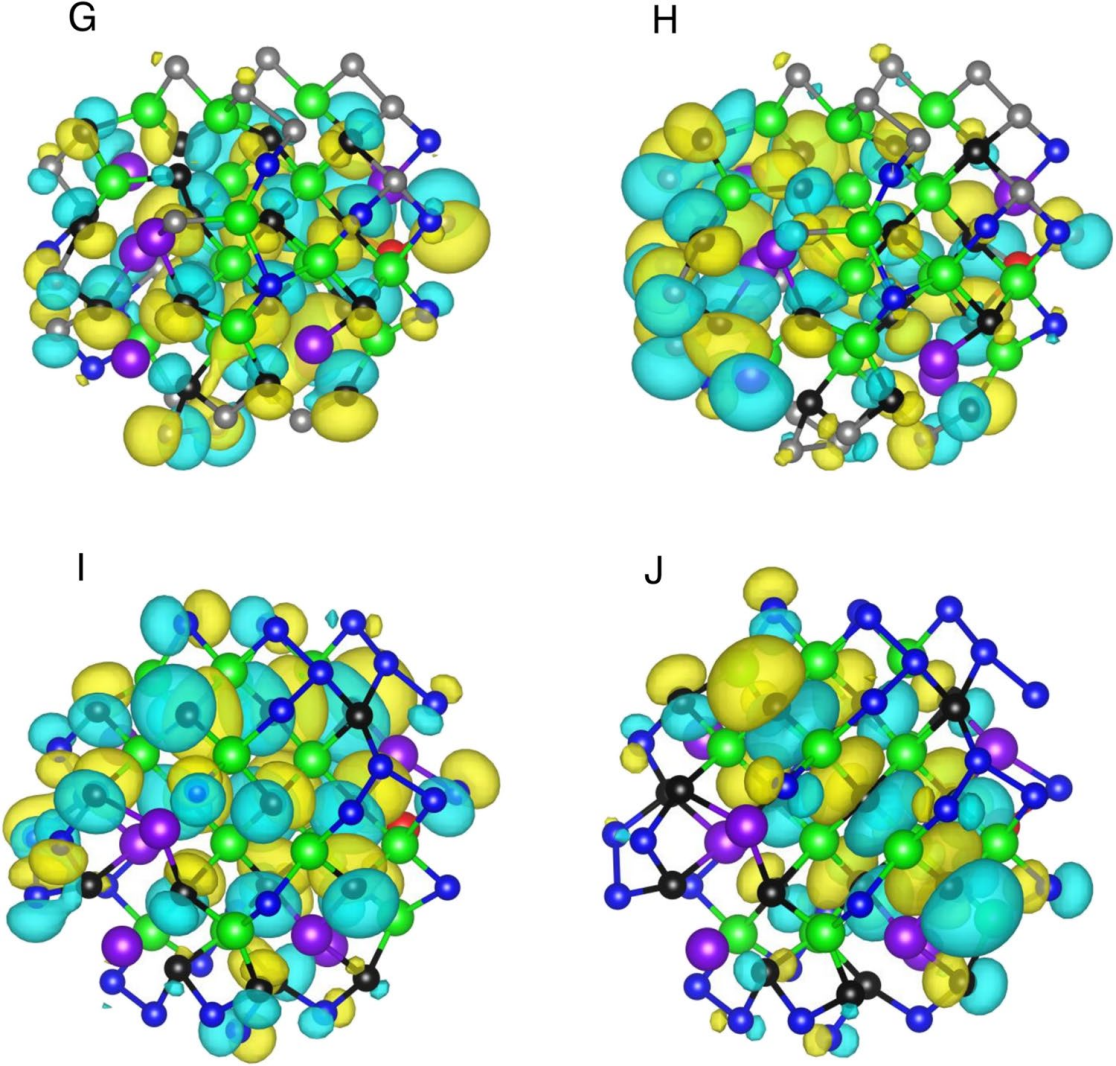
Composition-dependent HOMO and LUMO spatial distributions of GaInZnP/ZnSe_y_S_1-y_ core/shell QDs. **A** and **B** HOMO and LUMO for *y* = 0.00; **C** and **D** HOMO and LUMO for *y* = 0.25; **E** and **F** HOMO and LUMO for *y* = 0.50; **G** and **H** HOMO and LUMO for *y* = 0.75; and **I** and **J** HOMO and LUMO for *y* = 1.00. Isosurfaces are shown with positive and negative phases, and atom colours denote P, black; In, purple; Zn, red; Ga, green; S, grey; and Se, blue

Figure 5A–J shows the real-space isosurfaces of the HOMO and LUMO for GaInZnP/ZnSe_y_S_1-y_ QDs as a function of *y* from xTB calculations. The atom colours denote P as black, In as purple, Zn as red, Ga as green, S as grey, and Se as blue. This colour mapping was used to enable direct identification of the atomic sublattices that contribute most strongly to the frontier orbitals, based on the spatial concentration of the largest HOMO and LUMO isosurface lobes [32]. For *y* = 0.00, the HOMO in Fig. 5A is distributed over multiple atomic sites, with prominent lobes located across the framework defined by P black and the group III atoms, Ga green and In purple. The corresponding LUMO in Fig. 5B also exhibits multi-site character, but its dominant lobes are redistributed relative to the HOMO and are visually enhanced near peripheral regions that include the chalcogen atoms, S grey, together with neighbouring cation sites. This shows that, at *y* = 0.00, the occupied and unoccupied frontier orbitals are spatially distributed over different regions of the nanocrystal, indicating that they are associated with different local atomic environments. At *y* = 0.25, the HOMO in Fig. 5C maintains extended multi-site character, with substantial amplitude spanning regions containing P black and the cation network composed of Ga green and In purple. The LUMO in Fig. 5D displays a distinct spatial pattern, with several dominant lobes appearing closer to outer chalcogen-rich regions where Se blue is introduced alongside S grey. The largest LUMO lobes are not co-located with the principal HOMO lobes, indicating enhanced spatial differentiation between occupied and unoccupied frontier states upon partial Se substitution. For *y* = 0.50, the HOMO in Fig. 5E remains broadly distributed over the mixed cation framework, with visible amplitude across regions containing P black together with Ga green and In purple. The LUMO in Fig. 5F exhibits strong lobes that are more prominently associated with the mixed chalcogen environment, where S grey and Se blue coexist. Zn red sites are present in the vicinity of several LUMO lobes; however, the figure supports only that the unoccupied density is concentrated in regions that include these sites, not that Zn alone governs localisation. Overall, Fig. 5E and F shows that the composition-dependent redistribution is more pronounced for the LUMO than for the HOMO. At *y* = 0.75, the HOMO in Fig. 5G remains extended across multiple connected regions, with strong lobes frequently aligned with the P black and Ga green frameworks and adjacent In purple sites. The LUMO in Fig. 5H exhibits enhanced amplitude in peripheral regions enriched in Se blue, with several large lobes positioned around Se-containing sites and neighbouring cations. This indicates that, at higher Se fraction, the unoccupied frontier orbital places greater weight on Se-rich regions compared with lower y, while the HOMO remains more closely associated with the internal cation phosphorus framework. For *y* = 1.00, the HOMO in Fig. 5I continues to display multi-site delocalisation, with prominent amplitude distributed over regions containing P black and the cation network defined by Ga green and In purple. The LUMO in Fig. 5J shows dominant lobes concentrated around Se-rich regions, with several strong features located near the outer network where Se atoms are present. In this Se-rich limit, the LUMO remains delocalised over multiple atoms, yet its principal amplitude regions remain spatially distinct from those of the HOMO, preserving clear separation between occupied and unoccupied frontier states. Across Fig. 5A–J, both HOMO and LUMO are distributed over multiple atomic sites, demonstrating that neither frontier state collapses into a single site-localised orbital. Element-resolved inspection revealed a consistent qualitative contrast; the HOMO lobes are most prominent across regions defined by P black and the Ga green and In purple frameworks, whereas the LUMO lobes increasingly emphasise chalcogen-rich regions as Se blue progressively replaces S grey with increasing y. This composition-dependent redistribution of frontier orbital weight provides direct real-space evidence that ZnSe_y_S_1-y_ shell alloying modifies the spatial character of the occupied and unoccupied electronic edge states in GaInZnP-based core/shell QDs. The localisation analysis is thus based on orbital coefficient partitioning within the xTB framework and does not constitute a direct calculation of charge transfer or interfacial electron density redistribution. The procedure used for extraction of frontier orbital wavefunctions and generation of volumetric orbital data for visualisation is provided in Fig. S8.

### Onset energy and core/shell partitioning of frontier orbital density

**Fig. 6:**
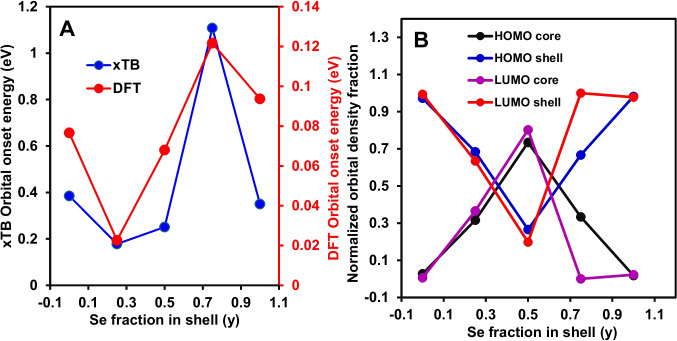
**A** Orbital onset energy as a function of Se fraction in the shell, *y* = 0.00, 0.25, 0.50, 0.75, 1.00, showing xTB and DFT benchmark comparison; **B** |Ψ|^2^ partitioned into core and shell regions for HOMO and LUMO as a function of y, normalized within the core/shell domain

Figure 6A presents the orbital onset energy as a function of Se fraction in the shell, *y*, from xTB analysis. The dependence is clearly non-monotonic. The onset decreased from 0.00 (relative to *y* = 0.75) to *y* = 0.25, increased slightly at *y* = 0.50 (relative to *y* = 0.25), reached a pronounced maximum at *y* = 0.75, and decreased again at *y* = 1.00. The magnitude of the increase at *y* = 0.75 is substantially larger than the incremental variations observed between *y* = 0.00, 0.25, and 0.50, thus establishing *y* = 0.75 as the most electronically distinct composition within the sampled range. The reduction from *y* = 0.75 to *y* = 1.00 indicates that the high onset at *y* = 0.75 is not simply a monotonic function of increasing Se content but reflects a composition-specific electronic response. For the DFT-based data, the absolute DFT onset energies were lower than the corresponding xTB values, which is consistent with the smaller frontier energy separations obtained from the present PBE-based DFT benchmark for these unpassivated models. However, for the purpose of compositional analysis, the more important result is that both methods identify the same two defining features of the trend, namely the minimum at *y* = 0.25 and the maximum at *y* = 0.75. This shows that the response of the frontier electronic structure to shell composition is not linear with increasing Se fraction. Instead, the data support a composition-specific electronic response, with the largest frontier state separation occurring at the intermediate composition *y* = 0.75 rather than at either compositional extreme. Accordingly, Fig. 6A shows that partial Se substitution modified the frontier orbital onset in a manner that depends on composition rather than on simple end-member interpolation. Within the sampled series, *y* = 0.75 is electronically the most distinct composition on the basis of orbital onset energy, whereas *y* = 0.25 gives the smallest onset in both approaches. This non-monotonic behaviour is important because it indicates that the frontier electronic structure evolves through a balanced alloying response across the shell composition range, rather than through a continuous one-direction shift with increasing Se content. It is important to emphasise that although orbital onset energy is defined relative to the HOMO reference at *E* = 0 eV, it does not represent a strict numerical equivalent of the HOMO–LUMO gap as shown in Fig. 3A. The HOMO–LUMO gap is obtained directly from the energy difference between the highest occupied and lowest unoccupied orbitals. In contrast, the orbital onset energy reflects the effective emergence of the unoccupied orbital manifold after energy alignment, which can be influenced by the distribution and spacing of near frontier states. In the present unpassivated QD models, the presence of surface localised states leads to closely spaced frontier orbitals, and the first accessible unoccupied level after alignment does not necessarily track the same compositional trend as the direct HOMO–LUMO gap. Thus, this difference explains why the maximum in Fig. 3A occurs at *y* = 0.50, while the orbital onset energy in Fig. 6A reaches a maximum at *y* = 0.75.

Figure 6B shows the partitioning of the frontier orbital density within the nanostructure region after normalisation of the integrated square of the wavefunction magnitude (|Ψ|^2^) contributions over the predefined core and shell domains. Under this definition, the core and shell fractions for each orbital sum to unity, allowing direct comparison of the relative localisation of the HOMO and LUMO as a function of shell composition. The dependence is non-monotonic for both frontier states. At *y* = 0.00, both orbitals are strongly shell weighted, with only minor core contribution, indicating that the frontier density is located predominantly in the shell region within the present partitioning scheme. At *y* = 0.25, the shell remains dominant for both HOMO and LUMO, although the core contribution increases, particularly for the LUMO, showing partial redistribution of the frontier density towards the interior region of the QDs. The largest change occurs at *y* = 0.50, where both frontier orbitals become core dominated. The HOMO showed a marked increase in core fraction relative to *y* = 0.25, and the LUMO shows an even larger core contribution. Within the present series, this composition therefore corresponds to the strongest relative shift of frontier density from shell-weighted to core-weighted character. At *y* = 0.75, the trend reverses, with the HOMO returning to shell-dominated character and the LUMO becoming almost entirely shell weighted. At *y* = 1.00, both frontier orbitals remain strongly shell weighted, indicating that the Se-rich limit is also characterised by dominant shell localisation in the renormalized core/shell partition. This distinct behaviour at *y* = 0.75 is consistent with a composition-specific redistribution of frontier orbital character rather than simple interpolation between the ZnS and ZnSe limits. At this composition, the frontier states show a pronounced return to shell-dominated localisation, while the orbital onset energy reaches its highest value, indicating that partial Se substitution produces the strongest perturbation of the near frontier electronic structure within the series. Thus, these results suggest that the *y* = 0.75 composition corresponds to a point of enhanced orbital reorganisation, in which the balance of S and Se contributions modifies the spacing and localisation of the frontier states more strongly than at the neighbouring compositions. The anomalous optical response observed at the same composition is therefore consistent with this frontier state redistribution, rather than with a simple monotonic effect of increasing Se content. In general, Fig. 6B shows that shell alloying does not produce a simple linear interpolation between the end members. Instead, the relative core/shell partitioning of both HOMO and LUMO changes nonlinearly, with an intermediate composition *y* = 0.50 at which both orbitals are more strongly associated with the core region, followed by renewed shell-weighted character at higher Se fraction. The workflow used for extraction of onset transition energy and evaluation of core-resolved and shell-resolved orbital density fractions is provided in Fig. S9. The use of unpassivated models provided a controlled framework for isolating composition-dependent effects, although surface-related states should be considered when interpreting absolute electronic and optical energies.

## Conclusion

A composition-controlled GaInZnP/ZnSe_y_S_1-y_ core/shell QDs series was analysed to resolve the effect of shell anion substitution on electronic structure and optical response under fixed structural conditions. The results show that both energetic stability and electronic properties vary nonlinearly with composition. S-rich shells were energetically favoured, while mixed compositions exhibited a pronounced deviation at *y* = 0.75, indicating non-ideal alloy behaviour. The frontier electronic structure was governed by composition-dependent modulation of orbital energy separation, with the HOMO–LUMO gap showing a non-monotonic trend and a maximum at intermediate composition. DOS-like distributions confirmed that the overall electronic framework was preserved, while DFT benchmarking supported that the nonlinear behaviour was intrinsic, with quantitative differences arising from the electronic structure method and surface-localised states. The optical response reflected this behaviour, with xTB sTDA showing redistribution of transition density and TDDFPT resolving a dominant absorption feature. Absorption onset energy varied nonlinearly across the series, with consistent compositional dependence between methods. The anomaly at *y* = 0.75, observed across energetic, electronic, and optical descriptors, indicated enhanced frontier state reorganisation within the mixed shell environment. Overall, shell alloying provided a direct route to tune electronic structure and optical response in core/shell QDs through controlled modification of orbital localisation and energy spacing.

## Supplementary Information

Below is the link to the electronic supplementary material.

ESM 1 (DOCX 154 KB)

## Data Availability

All data generated and analyzed in this study are presented within the manuscript and the Supplementary Information. Input files, processed datasets, and analysis scripts used for data generation and visualization are available from the corresponding author upon reasonable request.
