# Supplementary material for: Integrated xTB and simplified Tamm–Dancoff analysis of composition-dependent electronic structure in GaInZnP/ZnSeyS1-y core/shell quantum dots with DFT and TDDFPT benchmarking
Source: J Mol Model. 2026 May 8;32(6):172. doi: 10.1007/s00894-026-06728-1 (PMC13156133; doi:10.1007/s00894-026-06728-1)
Supplement: Supplementary file 1 — (DOCX 154 KB) [file 894_2026_6728_MOESM1_ESM.docx]

**Supplementary Information**

**Integrated xTB and simplified Tamm Dancoff analysis of composition dependent electronic structure in GaInZnP/ZnSe_y_S_1-y_ core/shell quantum dots with DFT and TDDFPT benchmarking**

Oluwasesan Adegoke ^1,^*, Ojodomo J. Achadu ^2^

*^1^* *Leverhulme Research Centre for Forensic Science, Faculty of Science, Engineering and Business, University of Dundee, Dundee, DD1 4HN, UK*

*^2^ School of Health and Life Sciences, and National Horizon Centre, Teesside University, TS1 3BA, Middlesbrough, UK*

* Correspondence

Oluwasesan Adegoke

[o.adegoke@dundee.ac.uk](mailto:o.adegoke@dundee.ac.uk)

^1^ Leverhulme Research Centre for Forensic Science, Faculty of Science, Engineering and Business, University of Dundee, Dundee, DD1 4HN, UK

**(A)**

Read optimised structure files (xtbopt.xyz) for each composition

Validate atomic coordinates and element identities

Assign composition label based on shell fraction (y)

Store structure for further processing

**(B)**

Export structure files in standard XYZ format

Ensure consistent atom ordering and formatting

Prepare files for external visualisation software

(C)

from ase.io import read, write

# Example: read optimised structure

atoms = read("xtbopt.xyz")

# Export for visualisation

write("structure.xyz", atoms)

**Fig. S1.** Procedure used for preparation of optimised QD structures for visualisation. (A) Extraction of atomic coordinates and element identities from xTB optimised geometry files for each composition. (B) Conversion of optimised structures into standard XYZ format for consistent use across visualisation tools. (C) Minimal self contained script illustrating structure reading and export using ASE.

**(A)**

Read total energies for all compositions

Map each structure to its corresponding Se fraction (y)

Sort data by composition

Identify minimum total energy across the series

Compute relative energy ΔE = E − E_min_

Convert energy to kcal/mol

Export processed dataset and plot ΔE as a function of composition

**(B)**

Read total energies for all compositions

Identify end member energies at y = 0.00 and y = 1.00

Perform linear interpolation between end members

Compute mixing deviation ΔE_mix_ = E − E_linear_

Convert energy to kcal/mol

Export processed dataset and plot ΔE_mix_ as a function of composition

(C)
import numpy as np

# Example input: columns = [y, energy_Eh]

data = np.loadtxt("energies.txt")

y = data[:, 0]

E = data[:, 1]

# Relative energy

Emin = np.min(E)

dE = (E - Emin) * 627.509

# Mixing reference

E0 = E[y == 0.0][0]

E1 = E[y == 1.0][0]

E_linear = (1 - y) * E0 + y * E1

dEmix = (E - E_linear) * 627.509

**Fig. S2.** Procedure used for evaluation of relative stability and mixing behaviour across shell compositions. (A) Calculation of relative stability based on total energy differences with respect to the lowest energy configuration in the series. (B) Calculation of mixing deviation using linear interpolation between end member compositions at y = 0.00 and y = 1.00. (C) Minimal self contained script illustrating relative energy and mixing deviation calculations from total energy input data.

**(A)**

Read orbital energies for each composition from xTB output

Read Kohn–Sham eigenvalues from DFT, NSCF calculations for each composition

Identify highest occupied and lowest unoccupied orbitals

Compute HOMO-LUMO gap as the energy difference

Map each structure to its corresponding Se fraction (y)

Sort data by composition

Perform identical HOMO-LUMO extraction for DFT eigenvalues using consistent energy reference

Export processed dataset and plot gap as a function of composition

**(B)**

Read molecular orbital energies from xTB electronic structure output

Read DFT eigenvalue spectra from NSCF calculations

Convert energies to electron volts

Align energies relative to HOMO reference

Align DFT spectra to their respective Fermi level or E_HOMO_ for direct comparison

Apply Gaussian broadening to each energy level

Sum contributions to obtain continuous distribution

Normalise distribution

Generate DFT DOS using dos.x for consistent spectral comparison

Export processed dataset and plot distribution for each composition

**(C)**

import numpy as np

# Example orbital energies in eV

energies = np.loadtxt("orbital_energies.txt")

# Example HOMO and LUMO values

ehomo = np.max(energies[energies <= 0.0])

elumo = np.min(energies[energies > 0.0])

# Fig. 3A style quantity

homo_lumo_gap = elumo - ehomo

# Fig. 3B to 3F style DOS like construction

energies_rel = energies - ehomo

grid = np.linspace(-6, 6, 2401)

sigma = 0.1

dos = np.zeros_like(grid)

for e in energies_rel:

dos += np.exp(-(grid - e)**2 / (2 * sigma**2))

dos /= len(energies_rel)

The same alignment and broadening procedure is applied to DFT eigenvalues to ensure consistent comparison between xTB and DFT spectral distributions.

**Fig. S3.** (A) Determination of HOMO-LUMO gap from orbital energy differences for each composition as a function of Se fraction (y) using xTB and DFT NSCF eigenvalue analysis. (B) Construction of DOS like distributions by applying Gaussian broadening to molecular orbital energies aligned relative to the HOMO level, alongside DFT DOS obtained from eigenvalue spectra and dos.x calculations. (C) Minimal self contained script illustrating orbital energy alignment and DOS like distribution generation with consistent numerical treatment across xTB and DFT datasets.

**Fig. S4.** TDDFPT absorption spectra of GaInZnP ZnSe_y_S_1-y_ core/shell QDs for y = 0.00, 0.25, 0.50, 0.75, and 1.00 plotted as a function of photon energy. Inset shows enlarged view of the main absorption peak region highlighting composition dependent shift in peak position and variation in intensity.

**(A)**

Read excitation energies and oscillator strengths from excited state output for both xTB sTDA and TDDFPT

Convert energies to wavelength if required

Define Gaussian broadening width appropriate for each method

Construct energy grid over relevant range

Apply Gaussian broadening to each excitation

Sum contributions to generate continuous spectrum

Normalise intensity for xTB sTDA spectra only, retain absolute oscillator strength scaling for TDDFPT spectra

Export processed dataset and plot spectrum

**(B)**

Read normalised spectrum data for each composition

Define intensity threshold for onset detection

Identify first energy where intensity exceeds threshold

Assign this value as absorption onset energy

Repeat for all compositions for both xTB sTDA and TDDFPT

Export onset energy as a function of composition

**(C)**

import numpy as np

# xTB sTDA spectrum construction from discrete excited states

# Example input: columns = [energy_eV, oscillator_strength]

data = np.loadtxt("excited_states.txt")

energy = data[:, 0]

osc = data[:, 1]

grid = np.linspace(min(energy), max(energy), 1000)

sigma = 0.2

spectrum = np.zeros_like(grid)

for e, f in zip(energy, osc):

spectrum += f * np.exp(-(grid - e)**2 / (2 * sigma**2))

# Normalise only for xTB sTDA figure presentation

spectrum_norm = spectrum / spectrum.max()

# TDDFPT onset extraction from processed spectrum

# Example input: columns = [energy_eV, intensity_raw]

tddft = np.loadtxt("plot_chi_spectrum.txt")

energy_tddft = tddft[:, 0]

intensity_raw = tddft[:, 1]

intensity_norm = intensity_raw / intensity_raw.max()

threshold = 0.10

onset_energy = energy_tddft[np.where(intensity_norm >= threshold)[0][0]]

**Fig. S5.** Procedure used for evaluation of optical absorption behaviour across shell compositions. (A) Construction of UV/Vis spectra by applying Gaussian broadening to excitation energies and oscillator strengths obtained from excited state calculations using xTB sTDA and TDDFPT. (B) Determination of absorption onset energy using a fixed intensity threshold applied consistently across compositions and methods. (C) Minimal self contained script illustrating xTB sTDA spectrum construction from discrete excitation energies and oscillator strengths, and TDDFPT absorption onset extraction from the processed spectrum using a fixed normalised intensity threshold.

**Fig. S6.** (A) Absorption onset energy and dominant absorption peak energy as a function of the Se fraction y in the ZnSe_y_S_1-y_ shell. (B) Distribution of optically allowed excited states plotted as oscillator strength (f) versus excitation energy for y = 0.00, 0.25, 0.50, 0.75, and 1.00. Inset o Fig. S1B: Projected view of the optically allowed excited states.

**(A)**

Read table containing composition (y), absorption onset energy, and peak energy

Validate required columns and remove invalid entries

Sort data by composition (y)

Export cleaned dataset for plotting

Plot absorption onset and peak energy as a function of composition

**(B)**

Read table containing excitation energies and oscillator strengths

Validate required columns and remove invalid entries

Filter excitation energies within defined energy window

Sort data by composition and excitation energy

Group data by composition

Plot oscillator strength stick spectra for each composition

Export processed dataset

**(C)**

import numpy as np

import matplotlib.pyplot as plt

# Example input: columns = [energy_eV, oscillator_strength]

data = np.loadtxt("excited_states.txt")

energy = data[:, 0]

osc = data[:, 1]

plt.vlines(energy, 0.0, osc)

plt.xlabel("Excitation energy (eV)")

plt.ylabel("Oscillator strength (f)")

plt.xlim(0, 4)

plt.tight_layout()

plt.savefig("excited_states.png", dpi=300)

plt.close()

**Fig. S7.** Algorithmic workflow used for optical descriptor extraction and excited state analysis. (A) Procedure for extracting absorption onset energy and peak energy as a function of shell composition (y) from processed excitation data. (B) Procedure for constructing oscillator strength distributions as a function of excitation energy for each composition. (C) Minimal self contained script illustrating the generation of oscillator strength stick spectra from excitation energy and oscillator strength input data.

**(A)**

Read electronic structure output and molecular orbital data

Identify highest occupied and lowest unoccupied orbital indices

Select corresponding orbitals from molecular orbital file

Generate volumetric wavefunction data on a three dimensional grid

Export orbital data in cube format for visualisation

**(B)**

# Example workflow for orbital extraction using Multiwfn

# Step 1: identify HOMO and LUMO indices manually from output

homo_index = 50

lumo_index = 51

# Step 2: run Multiwfn (interactive or scripted)

# Input: molden.input file

# Example commands inside Multiwfn:

# 5 -> molecular orbital analysis

# 4 -> output orbital to cube file

# homo_index -> select HOMO

# lumo_index -> select LUMO

# Output:

# HOMO.cube and LUMO.cube files

**Fig. S8.** (A) Procedure for extraction of frontier orbital wavefunctions. HOMO and LUMO orbitals were identified from electronic structure output, extracted from molecular orbital data, and exported as cube files for visualisation. (B) Minimal example workflow illustrating orbital selection and cube file generation. Orbital cube files were visualised using VESTA version 3.90.5a.

**(A)**

Read excited state transition data for each composition from xTB and DFT calculations

Identify lowest energy optically allowed transition

Extract corresponding excitation energy and wavelength

Map values to shell composition (y)

Sort data by composition

Export processed dataset and plot onset energy as a function of composition

**(B)**

Read orbital density data for HOMO and LUMO from xTB calculations

Integrate wavefunction density over core and shell regions

Normalize integrated values within the core and shell domain to ensure core and shell fractions sum to unity

Compute fractional contribution of core and shell

Map values to shell composition (y)

Export processed dataset and plot localisation fractions as a function of composition

(C)

import re

import csv

# Example DFT orbital onset extraction from NSCF output

text = open("y00.nscf.out").read()

m = re.findall(

r"highest occupied,\s*lowest unoccupied level \(ev\):\s*([-\d\.Ee+]+)\s+([-\d\.Ee+]+)",

text,

flags=re.IGNORECASE,

)

ehomo, elumo = map(float, m[-1])

orbital_onset_eV = elumo - ehomo

# Example assembly of xTB localisation fractions from table

with open("Table_Fig7_orbital_localisation_core_shell.csv") as f:

rows = list(csv.DictReader(f))

data = {}

for row in rows:

y = int(row["composition"].replace("y", "")) / 100.0

orb = row["orbital"].strip()

core = float(row["core_fraction"])

shell = float(row["shell_fraction"])

# Normalization within core and shell domain

total = core + shell

if total > 0:

core = core / total

shell = shell / total

data.setdefault(y, {})[orb] = {"core": core, "shell": shell}

**Fig. S9.** (A) Procedure for extraction of orbital onset energy from DFT NSCF output by reading the final highest occupied and lowest unoccupied levels for each composition and calculating E_LUMO_ - E_HOMO_. (B) Procedure for construction of orbital localisation trends from xTB derived core and shell fraction data for HOMO and LUMO as a function of composition. (C) Minimal workflow illustrating extraction of DFT orbital onset values from NSCF output and assembly of xTB orbital localisation fractions from the processed composition resolved table.
